# Weight, height, and midupper arm circumference are associated with haemoglobin levels in adolescent girls living in rural India: A cross‐sectional study

**DOI:** 10.1111/mcn.12908

**Published:** 2019-12-11

**Authors:** Anand S. Ahankari, Laila J. Tata, Andrew W. Fogarty

**Affiliations:** ^1^ Division of Epidemiology and Public Health University of Nottingham Nottingham UK; ^2^ HMF Research Halo Medical Foundation India; ^3^ Faculty of Health Sciences University of Hull Hull UK

**Keywords:** adolescent, anaemia, haemoglobin, India, Maharashtra

## Abstract

We aimed to explore the association of physical parameters with haemoglobin (Hb) levels to test the hypothesis that impaired physical development is associated with anaemia. A cross‐sectional survey study recruited adolescent girls (13 to 17 years) living in rural areas of Maharashtra state of India. Data were collected on physical parameters include height, weight, and midupper arm circumference (MUAC). Hb levels were measured using Sahli's haemometer. Linear regression was conducted to test the hypothesis. Data were collected from 1,010 girls on physical parameter and Hb levels. The majority of the adolescent girls were diagnosed with anaemia (87%). The regression analysis adjusted for age gave a significant association of Hb levels with all three variables (MUAC, weight, and height). Hb increased by 0.11 g/dl with an each centimetre of increase in MUAC (95% confidence interval, CI, [0.08, 0.15], *P* < .001). Each kilogram of increase in the body weight showed an increase in Hb levels (0.02 g dl, 95% CI [0.01, 0.03], *P* = .001). With an each centimetre of increase in height, Hb increased by 0.01 g dl (95% CI [0.00, 0.02], *P* = .022). There was a consistent association between three measures of somatic growth and anaemia in the study population. It is likely that life‐course exposures from conception onwards contribute to this, and the public health implications are that preventing anaemia is a challenge that requires a multifaceted interventional approach. Understanding the importance of the timing of these life exposures will help design interventions that can achieve optimal results.

**Key messages**

- Anaemia is a serious public health issue affecting a large number of Indian adolescent girls.
- We tested the hypothesis that impaired physical development is associated with anaemia.
- There was a consistent association between three measures of somatic growth and anaemia in the study population.
- It is likely that life-course exposures from conception onwards contribute to this.

## 1 INTRODUCTION

Anaemia is a global public health burden that affects half of preschool children, adolescent girls, and pregnant women. The World Health Organisation (WHO) has recognised anaemia as the second‐leading cause of disability and estimated that the number of anaemic cases worldwide is close to two billion (WHO, 2019a). India has the highest anaemic population that affects primarily pregnant women and adolescent girls. Out of 110 million Indian adolescent girls, between 68 and 98 million are estimated to be anaemic (WHO, 2019b). Anaemia, particularly during the early years of life, may lead to compromised cognitive development affecting school performance and education advancement (Mason et al., 2012), and it has negative influences on social and economic development. The cost of anaemia is estimated to be 6% of India's Gross Domestic Product (Sedlander, Rimal, Talegawkar, Yilma, & Munar, 2018; approximate annual loss of 135 billion USD). India also has a very high prevalence of malnutrition in young children and adolescents. A recent study from the north‐east region of India reported 54% of stunting in adolescents, which was higher in girls compared with boys (Pal, Pari, Sinha, & Dhara, 2017).

The Barker hypothesis suggests that diseases in adulthood are influenced by early life risk factors that have consequences through the rest of the life course (Barker, 1995). Somatic growth is particularly vulnerable and is influenced by environmental exposures until ending in early adulthood when the growth plates fuse. One important risk factor for anaemia is nutritional deficiencies of micronutrients such as iron, vitamin B12, and folate (WHO, 2019b). However, early life exposures may also be important in the development and maturation of the bone marrow. It is striking that even when adolescent females are treated with micronutrient supplement, a large proportion remain anaemic suggesting that other risk factors contribute to impaired haemoglobin (Hb) production in this population (FOGSI India, 2019).

We have used a cross‐sectional dataset to explore the association of physical parameters with Hb levels to test the hypothesis that impaired physical development is associated with anaemia.

## 2 METHODS

The Maharashtra Anaemia Study (MAS, Phase 1) was a cross‐sectional study designed to investigate risk factors associated with anaemia in adolescent girls (Ahankari, Myles, Fogarty, Dixit, & Tata, 2016). The study population consisted of 13‐ to 17‐year‐old girls living in 34 villages of Maharashtra state of India. The study area is known as one of the least developed regions in India having limited health and infrastructural facilities. Between April 2014 and June 2015, villages were visited with the aim of recruiting minimum 1,000 adolescent girls. No formal sample size calculation was performed as the Phase 1 project was designed as an initial feasibility study, and the outlined participant size was planned considering availability of time, funds, and scope of research. The study obtained ethical permissions from India and United Kingdom ethics committees, and written consents were obtained from each study participant and from their local guardian/parent/representative.

Data were collected on demographics and physical parameters, which were measured by the study team. Midupper arm circumference (MUAC) of the dominant arm was measured using a standard measuring tape at midpoint between the tip of the shoulder and the tip of the elbow. Height was measured on standing using a measuring scale. Weight was measured using the OMRON digital weighing machine. All measurement tools were validated on the first‐working day of each month as per the standard operating procedures (Ahankari, Myles, Tata, & Fogarty, 2019). Anaemia was diagnosed based on Hb levels assessed using the Sahli's haemometer device by trained medical staff. Sahli's method is a well‐established technique and widely used in rural India (Ahankari et al., 2016). The Sahli's method provides opportunity to estimate Hb instantly. Briefly, a blood sample is mixed with hydrochloric acid and the colour of the mixture is matched with comparator by addition of distilled water. The method is subjective; therefore, measurements generated during the research project were verified between two members of the project. Technicians involved in the MAS study had 4 years of laboratory experience and received necessary trainings to ensure quality of the investigation. Hb less than 12.0 g dl was characterised as anaemia in accordance with the WHO guideline, and investigations were conducted as per the standard operating procedures developed for this study (Ahankari et al., 2019).

The outcome of interest (Hb) was modelled as a continuous variable to study its relationship with height, weight, and MUAC. As there was a small number of parameters available with a high likelihood of colinearity, simple regression models that adjusted for age as a continuous variable were employed for each parameter separately to create three independent models. Goodness of fit was visually assessed using the residual versus predictor plots for each regression model. Analyses were performed using Stata 13.1 (StataCorp, College Station, Texas, USA).

### 2.1 Ethical statement

The study was approved by the Institutional Ethics Committee of the Government Medical College Aurangabad, Maharashtra, India (Reference number: Pharma/IEC/GMA/196/2014), and the Medical School Ethics Committee of the University of Nottingham, United Kingdom (Reference number: E10102013).

## 3 RESULTS

Data were collected from 1,010 adolescent girls from 1,023 who were invited to participate, giving a response rate of 99%. Study participants ranged from 13 to 17 years with a mean age of 14.9 years (standard deviation [*SD*]: 1.41). The majority of the adolescent girls were diagnosed with anaemia (87%), and of these, 17% had mild anaemia (Hb: 11.0 to 11.9 g dl), 65% had moderate anaemia (Hb: 8.0 to 10.9 g dl), and 5% had severe anaemia (Hb: ≤7.9 g dl). The Hb levels were normally distributed and ranged from 5.0 to 14.0 g dl (mean Hb: 10.1 g dl, *SD*: 1.3). MUAC ranged from 16 to 31 cm (mean 21.7, *SD*: 2.4). Height ranged from 124 to 169 cm (mean: 147.5, *SD*: 6.9), whereas mean weight was 38.6 kg (*SD*: 7.2).

The regression analysis adjusted for age gave a significant association of Hb levels with all three variables (MUAC, weight, and height; Table 1). Hb increased by 0.11 g dl with an each centimetre of increase in MUAC (95% confidence interval, CI, [0.08, 0.15], *P* < .001). On analysing Hb and weight, each kilogram of increase in the body weight showed an increase in Hb levels (0.02 g dl, 95% CI [0.01, 0.03], *P* = .001). The adjusted analysis also reported a significant association with height. With an each centimetre of increase in height, Hb increased by 0.01 g dl (95% CI [0.00, 0.02], *P* = .022). Residual plots reported random spread for all three independent variables (height, weight, and MUAC) around the axis line assessed in the study (Figure S1). As number of participants had similar height and MUAC measurements, thus residuals were grouped on the plot appearing as a pattern due to the similar readings.

**Table 1:** Characteristics of study population with regression analysis (*n* = 1,010 participants)

Characteristic	Participants (%)	Nonanaemic participants (%)	Anaemic participants (%)	Mean (standard deviation)	Age‐adjusted analysis^a^ (95% CI)	*P* value
Age in years						
13	207 (20.5)	25 (19.2)	182 (20.6)	14.9 years (1.41)	NA	NA
14	229 (22.6)	39 (30.0)	190 (21.5)			
15	199 (19.7)	26 (20.0)	173 (19.6)			
16	173 (17.1)	21 (16.1)	152 (17.2)			
17	202 (20.0)	19 (14.6)	183 (20.8)			
MUAC	1,010 (100)	130 (12.8)	880 (87.1)	21.7 cm (2.4)	0.11 [0.08, 0.15]	<.001
Weight	1,010 (100)	130 (12.8)	880 (87.1)	38.6 kg (7.2)	0.02 [0.01, 0.03]	.001
Height	1,010 (100)	130 (12.8)	880 (87.1)	147.5 cm (6.9)	0.01 [0.00, 0.02]	.022

Abbreviation: NA, not applicable.

a Beta coefficient with confidence intervals (CI) for difference in haemoglobin (grams/decilitre, g dl) as a continuous measure. Each coefficient is from a separate linear regression model (midupper arm circumference [MUAC], weight, and height, respectively) adjusted for age as a continuous variable.

## 4 DISCUSSION

These data collected from a rural population of India provided an opportunity to investigate the relationship of Hb levels with MUAC, weight, and height in adolescent girls. There is a positive association between Hb levels and each of the three physical parameters. This is consistent with the hypothesis that there is an association between Hb production and somatic growth, which could be a consequence of the accumulative impact of chronic exposures from early childhood onwards.

The MAS Phase 1 project has several strengths. The project is the first from Maharashtra state collecting data from a large rural difficult‐to‐access population that as a consequence is relatively neglected with regard to medical care and research. The response rate of 99% was high providing confidence that selection bias is unlikely. All data and samples were collected in a standardised manner, providing consistency across the study population.

Data on Hb were collected using Sahli's haemometer method, which is commonly used clinically in rural India, as it gives an immediate result in the rural environment where communication and transportation is challenging (Srivastava, Negandhi, Neogi, Sharma, & Saxena, 2014), although it is likely to have lower sensitivity and specificity compared with laboratory measurements of Hb. However, the Sahli's haemometer method provides a continuous epidemiological measure of Hb levels, and any imprecision or measurement error as a consequence of using this method will be systematic regardless of the growth parameter measurements and will thus have only resulted in weakening the associations observed in the regression analyses. We did not have data on advanced blood parameters such as serum iron, vitamins, and additional blood markers of adolescent girls and their mother. It is important to note that exposures during childhood and later in adolescence such as nutritional intake and access to anaemia public health services such as iron supplementation, onset of menstrual cycle, and medical history are of a major importance. Understanding these factors is also important to design optimal interventions for anaemia and at what stage in life they may be most effectively deployed.

MUAC, weight, and height can be considered as three different but complementary manifestations of somatic growth, which is inevitably influenced by composite experiences from conception to adolescence for our study population. Anaemia is a deficiency of red blood cells, which have a half‐life of approximately 120 days and hence can be regarded as an acute event or a chronic event depending on the context. The association that decreased somatic growth markers are associated with lower Hb measures is consistent with the Barker hypothesis that early life events impact on subsequent development and disease. However, as our data are cross sectional, they are only able to permit observation of associations rather than conclusions regarding casual relationships.

As regular nutritional supplements reduce the prevalence of anaemia in menstruating females (Low, Speedy, Styles, De‐Regil, & Pasricha, 2016), there is undeniably a nutritional deficiency that is contributing to impaired Hb production. However, the fact that low‐birthweight babies have an increased risk of anaemia at the age of 1 year (Ferri, Procianoy, & Silveira, 2014) and that many Indian adolescent females remain anaemic despite taking regular nutritional supplements (FOGSI India, 2019) allows us to speculate that impaired haematopoiesis may also coexist with nutritional deficiencies, possibly as a consequence of early life experiences (Zeeshan, Bari, Farhan, Jabeen, & Rathore, 2017). This is consistent with the observation that stunted women have smaller babies with higher rates of mortality (Low et al., 2016). Offspring of stunted women also have higher risks of stunted growth themselves resulting in shorter stature as adults (Addo et al., 2013). This suggests that there is a vicious cycle of impaired growth that is passed down through generations. It is likely that anaemia is part of these events, which form a syndrome of clinical consequences that may be challenging to prevent.

If the hypothesis that haematopoietic potential is determined from *early life* onwards is true, the public health implications are that preventing stunting and anaemia requires a multifaceted interventional approach. Cohort studies in these vulnerable populations are crucial to identify potential time windows where the stunting of somatic development and impaired Hb production are reversible or at least amenable to modifications. In addition, if stunted somatic growth is a consistently associated with a higher risk of anaemia, this could be used to identify adolescent females with a high risk of anaemia.

## CONFLICTS OF INTEREST

The authors declare that they have no conflicts of interest.

## CONTRIBUTIONS

The Maharashtra Anaemia Study Phase 1 was designed by Dr Andrew Fogarty, Dr Anand Ahankari, Dr Puja Myles, and Dr Laila Tata. This specific study hypothesis was designed by Dr Andrew Fogarty. ASA obtained the MAS Phase 1 data and conducted the analysis. All three authors (ASA, LJT, and AWF) participated in the manuscript preparation and approved the submission.

## Supporting information

Supplementary 1: Residual versus predictor plots
