# Supplementary material for: Weight, height, and midupper arm circumference are associated with haemoglobin levels in adolescent girls living in rural India: A cross‐sectional study
Source: Matern Child Nutr. 2019 Dec 11;16(2):e12908. doi: 10.1111/mcn.12908 (PMC7083455; doi:10.1111/mcn.12908)

Supplementary 1 Residual versus predictor plots

Figure 1a Mid upper arm circumference (MUAC)


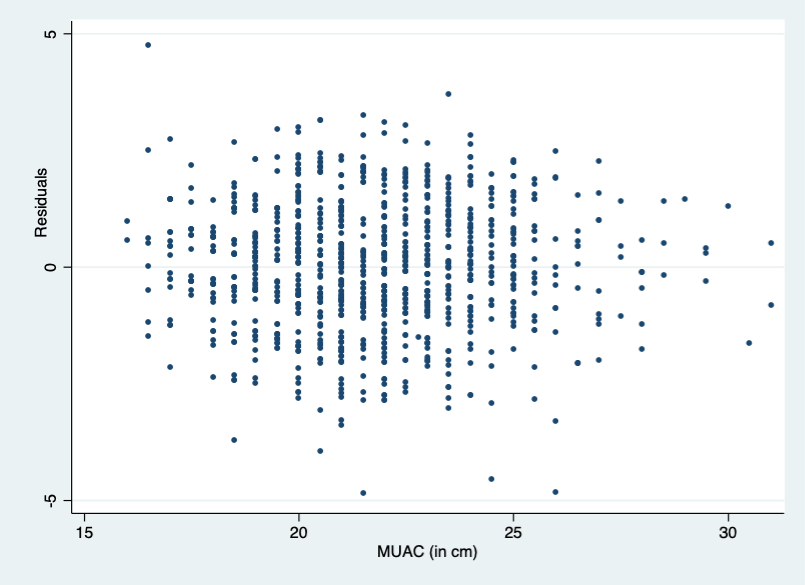


Figure 1b Weight


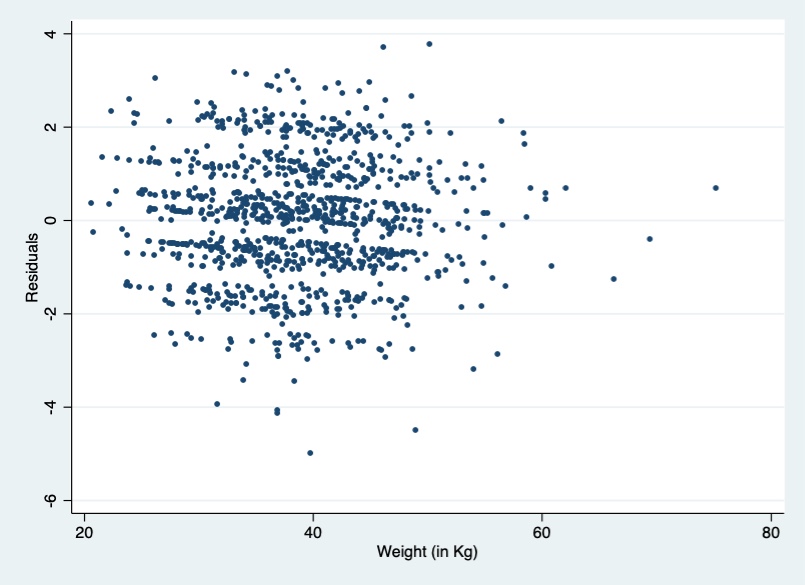


Figure 1c Height


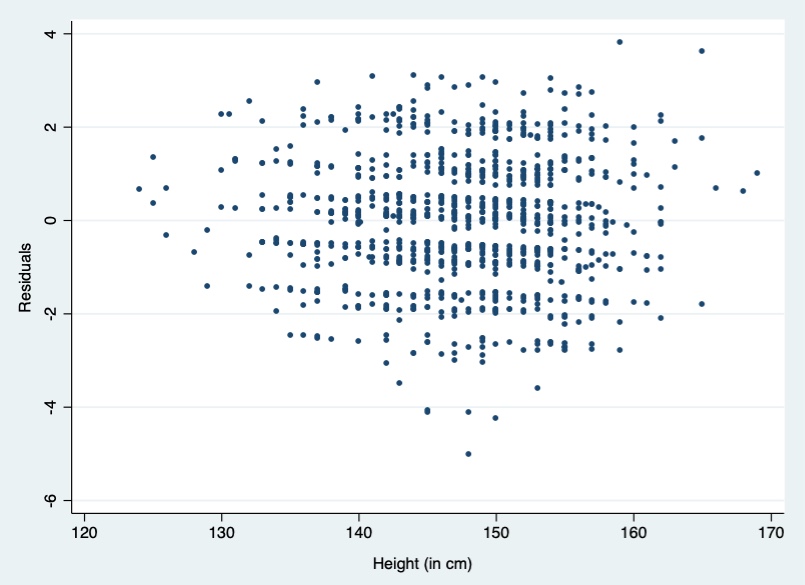

Supplement: Supplementary file 1 — Supplementary 1: Residual versus predictor plots [file MCN-16-e12908-s001.docx]
